# 

**Published:** 1843-05-27

**Authors:** 


// THE MEDICAL EXAMINER,

**And Retrospect of the Medical Sciences.**

EDITED BY

MEREDITH CLYMER, M.D.

Lecturer on the Institutes of Medicine, Physician to the Philadelphia Hospital, Blockley, Fellow of the College of Physicians, Philadelphia, etc. etc.

VOL. VI.] PHILADELPHIA, MAY 27, 1843. [No. 10.

